# Development of a Real-Time Tillage Depth Measurement System for Agricultural Tractors: Application to the Effect Analysis of Tillage Depth on Draft Force during Plow Tillage

**DOI:** 10.3390/s20030912

**Published:** 2020-02-08

**Authors:** Yeon-Soo Kim, Taek-Jin Kim, Yong-Joo Kim, Sang-Dae Lee, Seong-Un Park, Wan-Soo Kim

**Affiliations:** 1 Department of Biosystems Machinery Engineering, Chungnam National University, Daejeon 34134, Korea; kimtech612@gmail.com (Y.-S.K.); taekjin79@nate.com (T.-J.K.); 2 Convergence Agricultural Machinery Group, Korea Institute of Industrial Technology (KITECH), Gimje 54325, Korea; sdlee96@kitech.re.kr; 3 Research and Development Institute, Tongyang Moolsan Co. Ltd., Gongju 32530, Korea; psu@tym.co.kr

**Keywords:** agricultural tractor, tillage depth, draft force, sensor fusion, hardpan, plow tillage

## Abstract

The objectives of this study were to develop a real-time tillage depth measurement system for agricultural tractor performance analysis and then to validate these configured systems through soil non-penetration tests and field experiment during plow tillage. The real-time tillage depth measurement system was developed by using a sensor fusion method, consisting of a linear potentiometer, inclinometer, and optical distance sensor to measure the vertical penetration depth of the attached implement. In addition, a draft force measurement system was developed using six-component load cells, and an accuracy of 98.9% was verified through a static load test. As a result of the soil non-penetration tests, it was confirmed that sensor fusion type A, consisting of a linear potentiometer and inclinometer, was 6.34–11.76% more accurate than sensor fusion type B, consisting of an optical distance sensor and inclinometer. Therefore, sensor fusion type A was used during field testing as it was found to be more suitable for use in severe working environments. To verify the accuracy of the real-time tillage depth measurement system, a linear regression analysis was performed between the measured draft and the predicted values calculated using the American Society of Agricultural and Biological Engineers (ASABE) standards-based equation. Experimental data such as traveling speed and draft force showed that it was significantly affected by tillage depth, and the coefficient of determination value at M3–Low was 0.847, which is relatively higher than M3–High. In addition, the regression analysis of the integrated data showed an R-square value of 0.715, which is an improvement compared to the accuracy of the ASABE standard prediction formula. In conclusion, the effect of tillage depth on draft force of agricultural tractors during plow tillage was analyzed by the simultaneous operation of the proposed real-time tillage depth measurement system and draft force measurement system. In addition, system accuracy is higher than the predicted accuracy of ± 40% based on the ASABE standard equation, which is considered to be useful for various agricultural machinery research fields. In future studies, real-time tillage depth measurement systems can be used in tractor power train design and to ensure component reliability, in accordance with agricultural working conditions, by predicting draft force and axle loads depending on the tillage depth during tillage operations.

## 1. Introduction

Agricultural tractor performance is greatly affected by the draft force during tillage operations. The draft force is one of the most widely used indicators for evaluating the performance of agricultural tractors [1]. Studies analyzing and predicting the traction performance of agricultural tractors have used either the analysis method or the empirical method [2]. Of the two methods, the empirical method based on soil physical properties is relatively widely used because it can easily measure the mobility of the vehicle compared to the analysis method [3]. In general, many parameters affect the draft force of an agricultural tractor during tillage operations, such as soil texture, soil water content, traveling speed with slip ratio, and tillage depth, as well as soil strength in terms of cone index [4].

Many studies have been conducted on the factors affecting the draft force of agricultural tractors. In particular, various studies have been conducted on the effect of soil physical properties on draft force. A field experiment was conducted to measure the specific draught and energy use under different tillage implements and soil conditions [5,6]. The results showed that the specific draught was the lowest for the moldboard plow; conversely, it was the highest in chisel plows, and increased with decreasing soil water content. In another study, the specific draft estimation model for offset disc harrows was developed considering the front gang angle, cone index, and forward speed [7]; the results showed that a front gang angle of 35 degrees was required to minimize drafts. The effect of shank geometry and gear selection on tractor performance was analyzed during deep tillage operations [8]; the results showed that as the gear selection increased, the axle torque, draft force, and fuel consumption increased sharply. The study investigated the effect of soil texture and soil water content on the performance of agricultural tractors based on the USDA (U.S. Department of Agriculture) standard in upland fields [9]. The results showed that the soil texture parameter was between 1.4 and 1.5, which is higher than the guidelines set by the ASABE (American Society of Agricultural and Biological Engineers) standard, which prescribes 0.45–0.7 for the same draft force. In another study, an experiment was conducted to analyze the effect of implement surface coating on draft force during tillage operations [10]; the results showed that the modification of the furrower tines with an ultra-high molecular weight polyethylene (UHMW-PE) coating can significantly reduce the draft force. However, analytical studies on the influence of tillage depth on draft force and workload have been limited due to the difficulty of measurement.

Among the many factors that affect the loading of agricultural machinery, the tillage depth is key in the performance evaluation of the tractor because an actual paddy field has different soil properties according to the soil layers. The tillage depth affects the agricultural ecosystem and has a significant impact on crop yields and quality [11,12,13,14]. In theory, the target tillage depth is usually determined by the distribution of the hardpan. During plow tillage, the tractor attachment implement must penetrate deeper than the top hardpan depth during tillage. The formation of hardpan in the soil layer affects the soil compaction, which disturbs the root growth and permeation. Therefore, the plow tillage must be performed at the depth of the hardpan layer that is formed within the plow layer to determine that the tillage operation was performed at the appropriate depth. By analyzing the results of the cone penetration test, the depth of the cone index value within the plow layer is determined. This is assumed to be the average depth where the hardpan layer begins to be distributed, which is called the depth of the top hardpan [15,16]. 

Conventionally, tillage depth has been measured manually using a steel ruler. Some parameter studies including tillage depth related to tillage operation have been limited in some soil bin tests. Experiments were conducted to evaluate the performance of the soil strength profile sensor using soil bin and field data from different depth conditions [17]; the test results showed that the prismatic soil strength index was higher at locations with lower EC, lower water content, and greater bulk density. In other studies, experiments were performed to evaluate the effect of the design parameters of agricultural machinery on the draft force, rolling resistance, and soil compaction [18,19,20,21]. Although some studies have been conducted on the tillage depth performed in actual farm-land, it was not possible to conduct a precise test by roughly classifying the depth as shallow (0–0.15 m) and deep (0.15–0.3 m) or according to the soil layer [22,23]. In previous studies, tillage depth measurement was very labor-intensive and time-consuming. The bigger problem was that finding the average depth was not possible because continuous real-time measurement was not possible; the tillage depth was only available at some points [24]. As a result, it was not possible to analyze the effects of momentary changes in tillage depth, which would have been carried out under very rough test conditions. Until now, it has been difficult to measure tillage depth in real-time, so we were limited to analyzing the effect of tillage depth on draft force during tillage operations. So, the development and application of a real-time measurement system for tillage depth is needed. However, since it is difficult to verify the accuracy of continuous measurements during actual tillage, it is necessary to verify it using a formula for the interaction between the tillage depth and the draft force. Therefore, in this study, verification of the accuracy of the tillage depth measurement system was performed through an analysis of the effect of tillage depth on draft force during plow tillage.

The objectives of this paper are to propose improved measurement methods for tillage depth and to verify the accuracy of the developed measurement system through field experiments. The specific objectives were: (1) to develop a real-time tillage depth measurement system based on the sensor fusion method; (2) to continuously measure the tillage depth and the draft force during plow tillage; and (3) to confirm the accuracy of the real-time tillage depth measurement system by comparing it with the predicted draft force calculated by the ASABE standards equation.

## 2. Materials and Methods

### 2.1. Agricultural Tractor and Tillage Implement

The agricultural tractor (S07, Tong Yang Moolsan, Gongju, Korea) used in this study was equipped with a 78 kW-class engine with mechanical transmission type power shuttle and power shift (high, low). The 32 forward and 32 backward traveling speeds were determined by a combination of gear selection depending on the operation type. The tractor had an empty mass of 3985 kg, a total mass including all attached measurement system of 5260 kg and dimensions of 4.225, 2.140 and 2.830 m (length, width and height, respectively). The moldboard plow is mainly used in Korean paddy fields because it has better work stability than other types of plows during tillage operations. In general, the moldboard plow generates a high draft force during tillage operations [25]. Therefore, the moldboard plow is widely used for traction performance research. An eight-row moldboard plow (WJSP-8, WOONGJIN MACHINERY, Gimje, Korea) was used in this experiment. The specifications of the moldboard plow are shown in Table 1. 

### 2.2. Measurement of Soil Parameters

**Figure 1:**
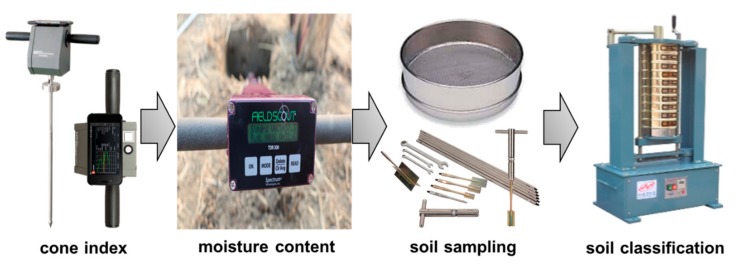
Measurement process of soil physical properties.

Repeated use of large farming machines, such as agricultural tractors, causes high pressure to be applied to the soil layer, which increases the thickness of the hardpan layer [26]. The formation of such a soil hardpan layer can affect the amount and distribution of the soil porosity, which can cause problems such as soil hardening or water drainage of farmland [27]. The soil physical properties found in the different soil layers depth during tillage can have a significant effect on mechanical strength. Among the various soil physical properties, cone index, soil water content, and soil texture are the major factors affecting the interaction between soil and agricultural machinery [28,29]. Therefore, precision measurement and analysis of soil physical properties are essential steps for studying the soil–machine interaction [30]. The cone index was measured using a cone penetrometer (FieldScout SC 900, Spectrum Technologies, Aurora, IL, USA), and the soil water content was measured using a soil water content sensor (FieldScout TDR 350, Spectrum Technologies, Aurora, IL, USA), respectively. Soil sampling was collected and classified using a soil sampler (HJD-1815, HEUNGJIN, Gimpo, Korea) and a ro-tap sieve shaker (HJ-2152, HEUNGJIN, Gimpo, Korea) for soil texture analysis based on the USDA standard method (Figure 1), which is the most widely used method in agricultural machinery research [31,32]. 

### 2.3. Tillage Depth Measurement

#### 2.3.1. Tillage Depth Measurement Principle

**Figure 2:**
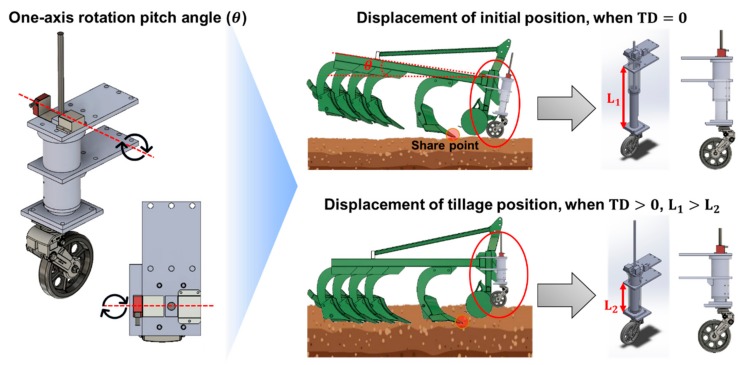
Measuring principle and its attachment position on the tillage depth measurement system.

The measuring principle and mounting position of the tillage depth measurement system are shown in Figure 2. In this study, a one-axis inclinometer was used to correct the vertical penetration depth measurement error caused by the tilting of the moldboard plow during tillage operations. Therefore, an inclinometer and optical distance sensor were attached on the left and right axis of the linear potentiometer, fixed to the center of the cylindrical jig, to use the pitch angle generated in the same axis. In addition, the tillage depth measurement system was attached to the front center of the outer moldboard frame to minimize the influence of the roll angle during tillage operations along the elevation of the non-tillage road surface.

The main measuring principle of this measuring system is the calculation of the relative vertical displacement difference of the share point of the moldboard plow, which is the end of the moldboard bottoms that are the first contact with the road surface and the deepest penetration during tillage operations. It is calculated by considering the difference between the vertical displacement (L_1_) and the pitch angle (θ_1_), measured as the share point of the moldboard, which comes into contact with the soil surface (TD = 0), and the vertical displacement (L_2_) and pitch angle (θ_2_) measured when the actual tillage depth occurs (TD > 0). Equation (1) was proposed for the measurement of tillage depth using the real-time tillage depth measurement system developed in this experiment:

(1) $$\mathrm{TD}=L_{1}\cos\theta_{1}-L_{2}\cos\theta_{2},$$

where TD (mm) is the tillage depth, L_1_ (mm) is the displacement of the real-time tillage depth measurement system measured by a linear potentiometer or optical distance sensor when the tillage depth is zero, θ_1_ (deg) is the pitch angle of the real-time tillage depth measurement system, which can be measured by an inclinometer when the tillage depth is zero, L_2_ (mm) is the displacement of the real-time tillage depth measurement system measured by linear potentiometer or optical distance sensor, and θ_2_ (deg) is the pitch angle of the real-time tillage depth measurement system and can be measured by an inclinometer.

#### 2.3.2. Development of a Real-Time Tillage Depth Measurement System

**Figure 3:**
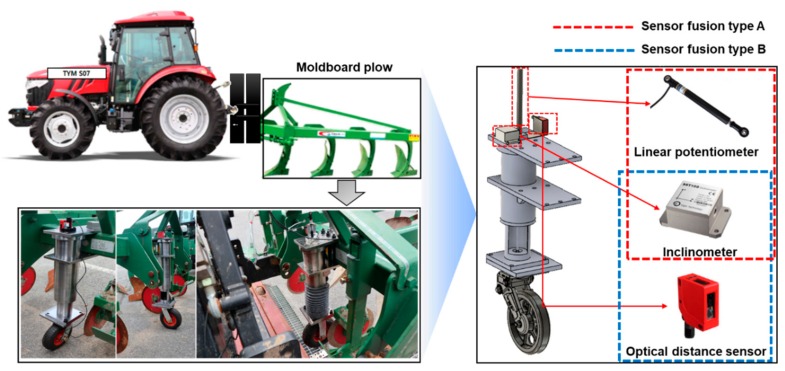
Configuration of the real-time tillage depth measurement system.

The detailed configuration of the real-time tillage depth measurement system is shown in Figure 3. The real-time tillage depth measurement system calculates the actual tillage depth using the vertical penetration depth and the pitch angle of the attached implement during tillage operations. The real-time tillage depth measurement system consists of a linear potentiometer (CLS1322, Active Sensors, Christchurch, UK) and optical distance sensor (ODSL 9/C6-650-S12, Leuze electronic, Owen, Germany) for measuring the vertical penetration depth of the attached implement, and a one-axis inclinometer (SST141, Vigor Technology, Shanghai, China) for measuring the tilt angle during tillage operations. The linear potentiometer has a measuring range of 0.025–0.35 m and an accuracy of <±0.05%. In addition, it has a durability rating of IP65 and can operate at up to 125 °C. The current type of optical range sensor used as a comparison with the linear potentiometer has a measuring range of 0.05–0.65 m and an accuracy of 1%. The one-axis inclinometer has a measurement range from 0 to 180° and an accuracy of ±0.05° at 25 °C. It has voltage outputs (0.5–4.5 VDC) proportional to the tilt angle of the attached implement. Measurement methods for real-time tillage depth measurement systems are divided into sensor fusion type A, which combines a linear potentiometer and an inclinometer, and sensor fusion type B, which combines an optical distance sensor and inclinometer, to select a more accurate measurement method through a comparison analysis after the accuracy verification test. The main body weight of the manufactured jig was designed using an alloyed steel material and electroplating was applied to prevent corrosion. The wheel attached below the main body is attached with a rubber wheel of 0.25 m diameter in order to maintain the vertical force without penetrating into the soil during the tillage operations.

### 2.4. Draft Force Measurement System

**Figure 4:**
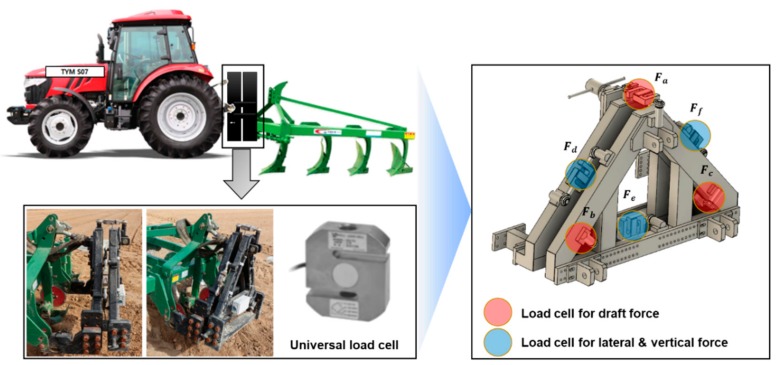
Configuration of the draft force measurement system.

In the field of off-road machinery research, a draft force measurement system consisting of six load cells is used for field tests of traction performance and to measure the draft, lateral and vertical forces of agricultural tractors [33,34,35]. The draft force measurement system is shown in Figure 4. The draft force measurement system consisted of six tensile compression-type universal type load cells (UU-T2, DACELL, Cheongju, Korea) to measure the forces that occurred during tillage. The tensile compression-type universal load cell used in this study has a rated capacity from 0–19.61 kN and the operating temperature was in the range (‒20 to +80°C). In addition, a GPS sensor (VBOX 3i, VBOX AUTOMOTIVE, Buckingham, UK) was attached to the center of gravity of the tractor to measure the traveling speed required for the comparative analysis using the actual measured draft force and the ASABE standards-based draft force prediction equation.

**Figure 5:**
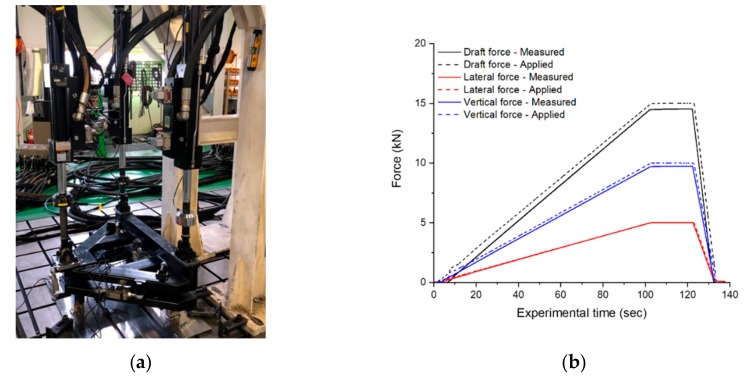
(**a**) Static load test for six-component load cell system; (**b**) test results of the static load test.

In general, static load tests are performed for each of the traction, lateral, and vertical forces to verify the accuracy of the six-component load cell system. A view of the static load test process with hydraulic actuator system is shown in Figure 5a. The static load test was conducted to analyze the system accuracy by repeatedly applying a constant force to a six-component load cell system by a linear hydraulic actuator. The system accuracy was analyzed by comparing the input load applied through the hydraulic actuator system and the load measured in the six-component load cell system. The static load test showed that all forces had a high accuracy of 97.4%–98.9%. The detailed static load test results for each type of force are shown in Figure 5b and Table 2.

In this experiment, only three load cells’ values for traction are considered [36]. The tillage depth was obtained using the following:

(2) $$D=F_{a}+F_{b}+F_{c},$$

where D (kN) is the draft force; F_a_, F_b_, and F_c_ are the forces (kN) measured from the load cells attached to the triangular frame edges of the six-component load cell system.

### 2.5. Test Procedure

#### 2.5.1. Soil Non-Penetration Test Using Two Sensor Fusion Type

The soil non-penetration test of the real-time tillage depth measurement system for accuracy verification was carried out at KITECH (the Korea Institute of Industrial Technology), Gimje-Si, Jeollabuk-do, Korea. The actual farm land where the tillage operations are carried out has typically uneven road surfaces, so it is very difficult to check the penetration depth in repeated tests. Therefore, a simple soil non-penetration test bed was made using square steel pipes of 0.2 m high for the accuracy verification of the real-time tillage depth measurement system. After placing the measuring tractor on the test bed, a soil non-penetration test for real-time tillage depth measurement system was carried out through 30 iterations of sensor fusion types A and B at each level of 0.05, 0.1, and 0.15 m. In this test, a comparison between sensor fusion types A and B was used to select a relatively precise method to be used in actual tillage operation. The system accuracy according to the sensor fusion method (through soil non-penetration tests) was obtained using the following equation:

(3) $${\mathrm{Acc}}_{\mathrm{TD}}=\left[1-\left(\left|{\mathrm{TD}}_{\mathrm{Ref}}-{\mathrm{TD}}_{\mathrm{Measured}}\right|/{\mathrm{TD}}_{\mathrm{Ref}}\right)\right]100,$$

where ${\mathrm{Acc}}_{\mathrm{TD}}$ is the accuracy of the real-time tillage depth measurement system developed in this study, ${\mathrm{TD}}_{\mathrm{Ref}}$ (mm) is the reference depth set as the target depth during the soil non-penetration test, and ${\mathrm{TD}}_{\mathrm{Measured}}$ (mm) is the depth measured by the real-time tillage depth measurement system during the non-penetration test.

#### 2.5.2. Field Experiments

The field experiments for continuous measurement of tillage depth and draft force were conducted in paddy fields located in Songsan-myeon, Dangjin-si, Chungcheongnam-do, Republic of Korea. The size of the paddy fields is 100 × 80 m and they are located at 36°55’48”N and 126°37’59”E on loam soil texture. The field tests were carried out in four-wheel mode with two gear stages (M3–low and M3–high) corresponding to the speed of the moldboard plow, and repeated three times for each gear stage. The soil physical properties’ measurement test was conducted before the actual tillage, and the main field soil information such as cone index, soil water content, and depth of soil layer was confirmed. The field experiment was conducted on a straight 100-m section and the tillage depth, traveling speed and draft force were measured simultaneously to analyze the effect of tillage depth on draft force at each gear selection.

### 2.6. Analysis Method

To analyze the effect of tillage depth on draft force, it is necessary to select the range of tillage depths in which the tillage operation is carried out. In this study, the data measured through the field tests were classified according to the tillage depth, and the influence of the tillage depth on the draft force during tillage operations was analyzed. It is difficult to verify the accuracy of the real-time tillage depth measurements system during field experiment with continuous tillage. Therefore, this study used an analytical method to compare the measured values using tillage depth and the draft force measurement system and the predicted draft force using the equation provided by the ASABE standards [37]. The dimensionless soil texture adjustment parameter F_i_ of the test field used in the draft force prediction equation was selected according to a soil texture analysis based on the USDA soil texture classification method. In this study, a method for evaluating the system accuracy through an error analysis between the measured and predicted values was used [38,39]. In order to verify the accuracy of the real-time tillage depth measurement system, linear regression statistics were performed through statistical software IBM SPSS Statistics (SPSS 25, SPSS Inc., New York, USA) to compare the measurement accuracy between the measured draft force and the predicted draft force under actual tillage conditions. In addition, a pre-analysis was conducted to determine under which conditions, such as tillage depth, gear selection, and traveling speed, the analysis should be performed to produce the highest accuracy when compared with the ASABE standard prediction equation. In detail, the system error and accuracy were analyzed through the coefficient of determination and root-mean-square error (RMSE) results, derived by linear regression, to see if the plow tillage under these particular conditions of tillage depth, gear selection, and traveling speed shows high accuracy in comparison with the predicted values using the ASABE standard prediction equation. The coefficient of determination and root-mean-square error through regression analysis were obtained through the following equations:

(4) $$R^{2}=\frac{\sum_{i}{\left({\hat{y}}_{i}-\bar{y}\right)}^{2}}{\sum_{i}{\left(y_{i}-\bar{y}\right)}^{2}}=1-\frac{\sum_{i}{\left(y_{i}-{\hat{y}}_{i}\right)}^{2}}{\sum_{i}{\left(y_{i}-\bar{y}\right)}^{2}}$$

where R^2^ is the coefficient of determination of the regression equation, and $y_{i}$ is the y value for observation i, and $\bar{y}$ is the mean of y value, and $\hat{y}$ is the predicted value of y for observation i.

(5) $$\mathrm{RMSE}=\sqrt{\frac{\sum {\left(Y-\;Y\prime \right)}^{2}}{N}}$$

where RMSE is the root-mean-square error, Y is the measured value, Y’ is a predicted value, and N is the number of pairs of values. The numerator is the sum of squared differences between the measured value and the predicted value.

Conventionally, a draft force during tillage operation is estimated using the following simplified prediction:

(6) $$D=F_{i}\left[A+\mathrm{BS}+{\mathrm{CS}}^{2}\right]\mathrm{WT}$$

where D (kN) is the draft force of attached implement, F_i_ is the dimensionless soil texture adjustment parameter with different values (F_i_ = 1 for fine, 0.7 for medium and 0.45 for coarse textured soils), A, B, and C are machine-specific parameters determined by the implement type, S (km/h) is the traveling speed of agricultural tractor, measured by GPS, W (m) is the implement width, and T (cm) is the tillage depth.

## 3. Results and Discussion

### 3.1. Soil Non-Penetration Test

**Figure 6:**
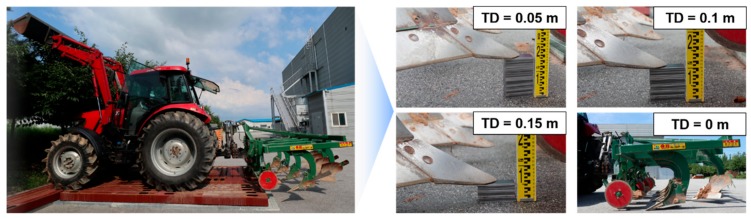
Soil non-penetration test of the real-time tillage depth measurement system.

The overall soil non-penetration test procedure for accuracy verification using a real-time tillage depth measurement system is shown in Figure 6. A simple repeated soil non-penetration test was performed to verify the tilling depth measurement system with two types of sensor fusion methods. The accuracy verification test was carried out after placing the main body of the tractor on the test bed and placing a steel block at the lower point of the share point to limit the lowering range of the three-point hitch according to the target tillage depth. In the case of a target tillage depth of 0.05 m, sensor fusion type A showed 97.9% measurement accuracy with a RMSE of 0.00105 m, and sensor fusion type B showed 89% measurement accuracy with a RMSE of 0.0055 m. In the case of a target tillage depth of 0.1 m, sensor fusion type A showed 96.7% measurement accuracy with a RMSE of 0.00327 m, and sensor fusion type B showed 90.4% measurement accuracy with a RMSE of 0.0096 m. Sensor fusion type B showed its highest measurement accuracy at 0.1 m target tillage depth, but was still 6.3% lower than sensor fusion type A. In the case of a target tillage depth of 0.15 m, sensor fusion type A showed 99.2% measurement accuracy with a RMSE of 0.00106 m, and sensor fusion type B showed 87.3% measurement accuracy with a RMSE of 0.01895 m. Sensor fusion type A showed the highest accuracy, while sensor fusion type B showed the lowest accuracy at 0.15 m, which is the tillage depth that occurs most often during actual tillage operations. Although the overall tendency was similar because pitch angle data from the same inclinometer were used, sensor fusion type A was 6.3%–11.9% more accurate than sensor fusion type B. Sensor fusion type A was fixed at the end of the shaft extended from the linear potentiometer directly to the bottom of the jig main body, so the error caused by lateral and vertical vibrations was very small even in the case of large vibrations during actual tillage operations. However, sensor fusion type B tended to detect longer distances than the actual distance, as the distance from the optical distance sensor to the target surface was changed temporarily due to the vibration of the attached implement. From these results, it seemed that sensor fusion type A is more suitable for measuring the tillage depth during tillage operations in a severe farming environment. The specific accuracy verification test results of the tillage measurement system are shown in Table 3.

### 3.2. Soil Properties

**Figure 7:**
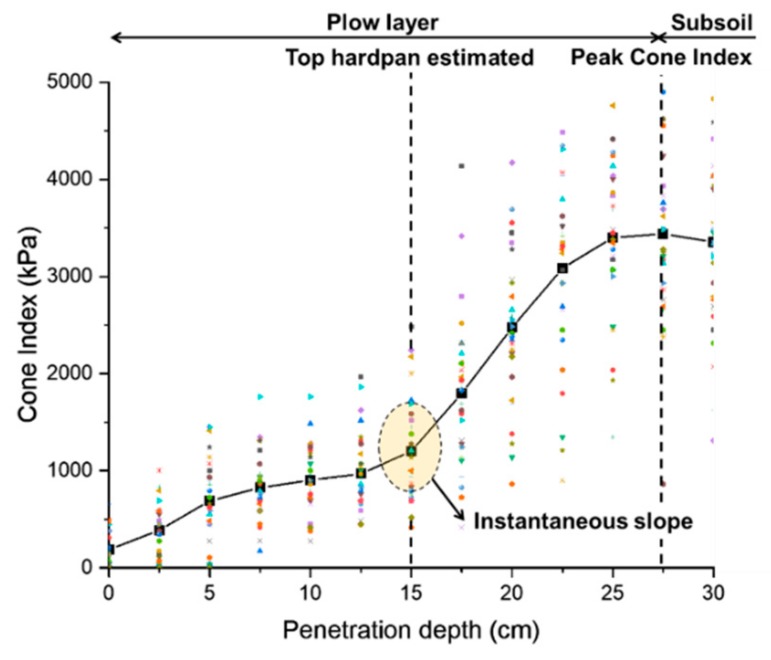
Results of average cone index according to penetration depth.

The average soil water content and average cone index by penetration depth were analyzed through 30 measurements at the test site. The average soil water content was 33.8% and the average cone index results at each tillage depth are shown in Figure 7. Layer 0–0.05 m depth showed an average cone index value of 424.2 kPa and layer 0.05–0.15 m showed an average cone index value of 901.2 kPa. An instantaneous slope occurred at the penetration depth of 0.12–0.15 m, and the peak cone index value was seen at a penetration depth of approximately 0.27 m. In the 0.15–0.27 m section, the average cone index value increased rapidly, with an average cone index value of 2567.9 kPa. Based on these results, the depth estimated to be the hardpan starting depth among the plow layers is about 0.12–0.15 m, and the estimated starting depth of the subsoil layer is about 0.27 m [16]. Therefore, the data measured in the field test were analyzed for a tillage depth range of 0.14–0.19 m, which was determined to be performed at the hardpan depth. As a result of soil texture analysis, the sampled soil consisted of 34% sand, 48% silt, and 18% clay and was classified as loam according to the USDA soil classification standards. The soil texture adjusted parameter (F_i_) used in Equation (6) was defined as 0.7 because it was classified as a medium texture based on the USDA soil texture classification method [40,41].

### 3.3. Analysis of Field Experiment Data

#### 3.3.1. Tillage Depth

**Figure 8:**
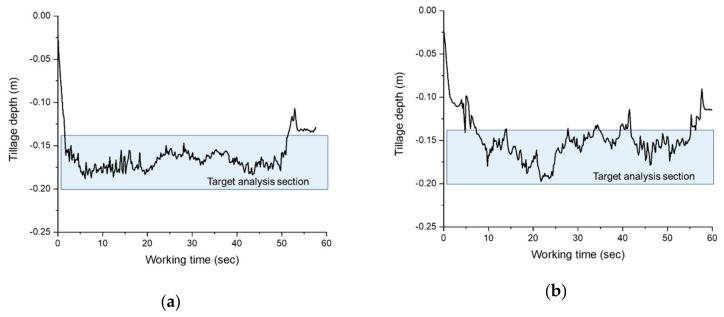
Measured tillage depth during plow tillage at (**a**) M3–low; and (**b**) M3–high.

The tillage depth measured by a real-time tillage depth measurement system is shown in Figure 8. The mean tillage depth at M3–low was 0.168 m, which indicates that the plow tillage was performed stably in the hardpan layer, the target tillage depth range. As a result of analyzing all sections including the plowshare penetration, Figure 8a shows that, in most of the working sections, over 86% of the measured tillage depth data were distributed in the hardpan layer. The mean tillage depth at M3–high was 0.157 m, which was 6.1% shallower than M3–low, and the variation was relatively large, indicating an unstable tillage depth. As a result, the graph waveform of the overall tillage depth showed a large deviation according to the working path, as shown in Figure 8b. Although the maximum tillage depth was deep, the ratio of the hardpan layer to the overall working path was about 75%, which was lower than that of M3–low. Descriptive statistics of measured tillage depth at each gear selection during plow tillage are shown in Table 4. Plow tillage was carried out on the same three-point hitch for a tillage depth section of 0.16–0.2 m, but the average tillage depth at each gear stage was different. It is believed that the different gear stages were affected by the occurrence of different soil resistance and draft force in the same tillage depth during tillage. Descriptive statistics showed that the standard deviation of measured tillage depth was about 0.01 m on average. Therefore, the field test data were grouped by 0.01 m to remove any accidental irregularities in the test and analyzed using the group frequency distribution method to clearly analyze the effect of tillage depth on dependent variables such as traveling speed and draft force.

#### 3.3.2. Traveling Speed

The theoretical speeds of the two gears selected in the field experiments were 7.09 and 8.43 km/h, which were suitably matched to the working speed range of the attachment plow implement. The average traveling speeds of the gear selections were 5.69 and 5.58 km/h. The traveling speed of the overall mean showed similar values, but different trends were analyzed according to the tillage depth. The overall average traveling speed at M3–low was higher than the M3–high gear stages. As the depth increased, the traveling speed decreased by 4.5% at 0.19 m tillage depth compared to the overall average traveling speed of M3–low. The traveling speed at M3–low was 5.44 km/h at the deepest tillage depth (0.19 m), which was 4.3% lower than the fastest traveling speed at 0.16 m. The M3–high gear selection with the fastest theoretical speed had a slightly lower average traveling speed than the M3–low. In the case of the M3–high, as the tillage depth deepened to 0.19 m, it tended to sharply decrease (by 26.6%) the average traveling speed. This seems to be affected by the large soil resistance caused by the faster speed in the tillage depth section deeper than 0.16 m. It is also believed that this influenced the uneven tillage depth. The detailed results of traveling speed according to tillage depth with ANOVA analysis using DMRT (Duncan’s multiple range test) are summarized in Table 5.

#### 3.3.3. Draft Force

**Figure 9:**
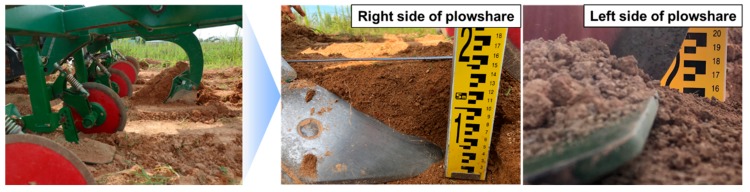
Manually measured tillage depth in test field.

Field experiments of the real-time tillage depth measurement system using sensor fusion type A in the paddy fields are shown in Figure 9. In accuracy tests carried out on actual paddy fields, the measurement error was 0.0042 m between the monitored tillage depth of 0.1602 m and the measured tillage depth of 0.156 m. The measurement accuracy was 97.3%, which was similar to the accuracy of the soil non-penetration test.

As a result of measuring draft force and tillage depth during tillage, the overall average draft force for each gear was similar to 32.66 ± 1.94 and 32.78 ± 2.44 kN, respectively. However, the draft force according to the tillage depth showed a difference in tendency for each gear stage. In the case of M3–low, the average draft force increased 7.2% as the tillage depth increased from 0.14 to 0.19 m. The average draft force in the 0.14–0.16 m range tended to be similar, with a difference of 0.7%–1.2% compared to the M3–high, but up to 7% lower in the deep tillage depth of 0.17–0.19 m. In addition, the draft force at M3–low increased 7.2% as the tillage depth increased from 0.14 m to 0.19 m. Plow tillage in M3–low was generally stable with a constant tillage depth, but draft force tended to be affected by tillage depth. As the tillage depth increased from 0.14 m to 0.19 m, the average draft force of M3–high also increased by 13.3%. The average draft force at M3–high was up to 7% higher than that of M3–low, which was considered to be affected by the high reduction of traveling speed at the deep tillage depth range. Although the draft force increased as the tillage depth deepened, it showed relatively unstable tillage depth compared to M3–low. The detailed results of measured draft force by draft force measurement system according to tillage depth with ANOVA analysis using DMRT are summarized in Table 6.

## 4. Discussion

**Figure 10:**
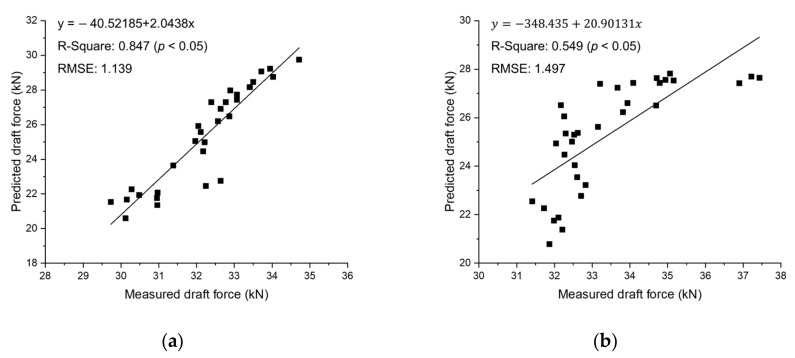
Results of linear regression between the measured draft force and the predicted values based on ASABE standard equation at (**a**) M3–low; and (**b**) M3–high.

The accuracy of the real-time tillage depth measurement system was analyzed during plow tillage by comparing the measured draft force through the field test and the predicted value using the ASABE standard equation. The results of a linear regression using a F_i_ value of 0.7 for the two gear stages are shown in Figure 10. The accuracy of the tillage depth measurement system, defined as the coefficient of determination and indicated by the regression analysis between measured and predicted draft force values, tended to vary depending on the working conditions during plow tillage. In M3–low, results of linear regression showed a high coefficient of determination value of 0.847 and low RMSE (Root Mean Square Error) of 1.139, as shown in Figure 10a. In addition, the coefficient of determination value was close to 1 in the deep tillage depth section where high draft force is generated. The high accuracy in the deep tillage depth range is influenced by the fact that the traveling speed is not significantly reduced even in the deep tillage depth section in M3–low. On the other hand, results of linear regression at M3–high showed lower system accuracy than M3–low with a coefficient of determination of 0.549 and RMSE of 1.497, as shown in Figure 10b. This has a relatively faster theoretical speed compared to M3–low at M3–high, and is believed to have been affected by the rapid deceleration of the traveling speed due to the large soil resistance at the deep tillage depth section.

**Figure 11:**
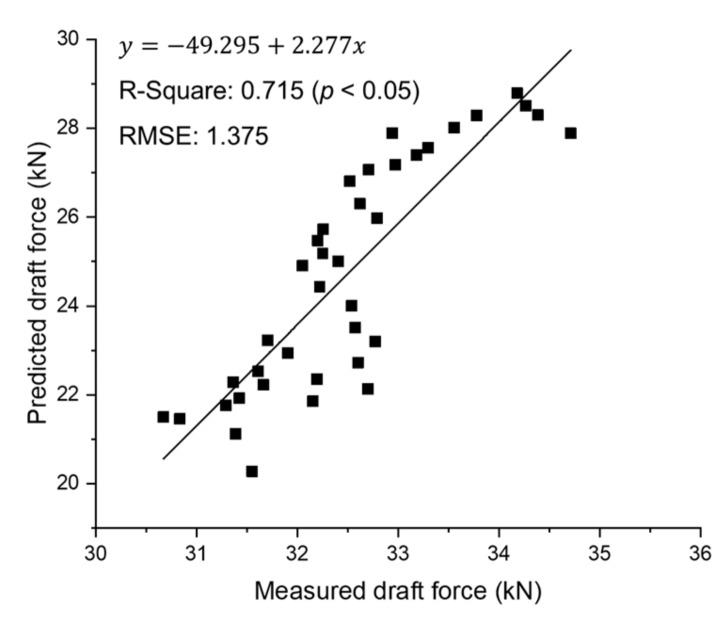
Results of linear regression between the measured draft force and the predicted values based on the ASABE standard equation using integrated data of two gears.

In actual plow tillage, the farmer could use both gears, so the measured draft force data from the two gears used in the field test were integrated and analyzed for further linear regression. As can be seen from Figure 11, it shows a coefficient of determination of 0.715 and RMSE of 1.375, which is smaller relative error than M3–high but larger than M3–low. The accuracy of the real-time tillage depth measurement system, which shows an R-Square value of 0.715, is improved compared to the accuracy of the ASABE standard prediction equation with an error range of ±40%, and it can be used in various studies on tillage depth and draft force. The deviation between measured and prediction can be attributed to: (1) the limitation caused by roughly distinguishing only three dimensionless soil texture adjustment parameter (F_i_) values, even though the soil texture is classified into various types, (2) use of different types of agricultural tractors and implements, and (3) soil properties differences present during the field experimental process (e.g., inconsistence of moisture content, hardpan, and soil surface roughness between working paths). Overall, the deeper the cultivation depth in both gear stages, the smaller the difference between the predicted and measured values, resulting in improved system accuracy. It is believed that the system accuracy is reduced in deep tillage depth conditions, where the traveling speed is reduced due to soil resistance. In conclusion, the accuracy of the draft force prediction using the real-time tillage depth measurement system was greatly influenced by the traveling speed and soil penetration depth of the attachment implement. Therefore, the tillage depth should be considered when analyzing the performance agricultural tractors. It is very important to know the depth at which the tillage operation was performed to ensure tractor design reliability. Through this study, system accuracy with an R-square value of 0.715 could be obtained using the developed real-time tillage depth measurement system. This system is expected to be of great help for future research on traction force prediction according to the working conditions.

## 5. Conclusions

In this study, a real-time tillage depth measurement system was developed for continuous measurement during plow tillage to analyze the effect of tillage depth on draft force using a sensor fusion method. The real-time tillage depth measurement system developed in this study selected the sensor fusion type A (linear potentiometer, inclinometer), suitable for actual tillage operations according to a soil non-penetration test. The selected sensor fusion method was used to continuously measure tillage depth and draft force during plow tillage. Finally, the accuracy of the real-time tillage depth measurement system was evaluated by comparing the measured draft force and predicted draft force based on the ASABE standard. The main results of this study are as follows.

(1) The real-time tillage depth measurement system was developed through sensor fusion consisting of a linear potentiometer, an optical distance sensor, and an inclinometer. A simple soil non-penetration test bed was constructed considering the actual tillage depth of moldboard plow. As a result of the accuracy verification test, sensor fusion type A showed a 2% measurement error, which was 9% lower than the sensor fusion type B, using the optical distance sensor and the inclinometer, which had an 11% measurement error. In addition, sensor fusion type A showed only about 3% measurement error in actual moldboard plow operations. Therefore, sensor fusion type A is a suitable method for measuring the tillage depth during actual tillage operations. In addition, the system can only be measured with relative vertical displacement, which makes it possible to use it for any type of tillage operation.
(2) To verify the accuracy of the real-time tillage depth measurement system in the plow tillage, a comparative test between the measured draft force and the predicted values based on the ASABE standard equation was conducted. In addition, soil texture analysis of the test field was performed to select F_i_ (dimensionless soil texture adjustment parameter), a key factor used in the draft force prediction. As a result of the soil texture analysis, the soil of the test field was classified as loam, and it was confirmed that the F_i_ value of 0.7 should be used when using the draft force prediction equation of ASABE standard as the soil texture of the medium texture group.
(3) Field test results showed that the overall average traveling speed and draft force values of the two gear stages were similar, but are greatly affected by the tillage depth. Overall, the higher the gear selection, the deeper the tillage depths, which will result in greater soil resistance, slowing the traveling speed while increasing the average draft force. The regression analysis for system accuracy analysis showed that the deeper the tillage depth in each gear, the closer the coefficient of determination was to 1. Specifically, the lower the gear selection and the deeper the tillage depth during plow tillage, the higher the coefficient of determination as a result of the regression analysis of the measured values. This is thought to be influenced by the relatively rapid decrease in traveling speed due to greater soil resistance in the deep tillage depth section of the high gear stage M3–high. Therefore, in the case of no reduction in traveling speed due to soil resistance even in the deep tillage depth section during plow tillage, it was seen that the deeper the tillage depth and the greater the draft force, the higher the accuracy of the draft force prediction using the real-time tillage depth measurement system.

Although some aspects need to be improved, this tillage measurement method is judged to have a wide utilization range for research into the effect of the tillage depth on the loads of agricultural tractor during tillage operations. It is judged that this will enable the study of the effects of improved measurement precision and machine reliability on the local soil, subject to future study and testing, and that it will be useful for the study of traction performance and design load of tractors considering the agricultural working environment.

In conclusion, the effect of the tillage depth on the draft force of agricultural tractors during moldboard plow operations was confirmed with the proposed measurement system configuration. The results of this study will be useful for field test condition setting and data analysis of the measured workload to be used for the optimal design of agricultural tractors. In future studies, the soil–machine parameters according to tillage depth will be analyzed by field experiments when attaching other tillage implements, and a discrete element method model for agricultural machinery will be developed to conduct a parameter simulation on the factors affecting the design load of agricultural tractor and model verification by field demonstrations.

## Figures and Tables

**Table 1:** Specification of the moldboard plow used in this study.

Item	Specification
Mass (kg)	790
Length × Width × Height (m)	2.8 × 2.15 × 1.25
Required power (kW)	67–89
Working depth (m)	Up to 0.2
Working speed (km/h)	5–8
Share type	Gunnel-type/Plain coulter with spring

**Table 2:** Specific result of the static load test for six-component load cell system.

Item	Draft	Lateral	Vertical
Applied force (kN)	14.7	9.8	4.9
Measured force (kN)	14.5	9.7	5
Accuracy (%)	98.9	99.2	97.4

**Table 3:** Results of soil non-penetration test according to sensor fusion type.

Target Tillage Depth (m)	Sensor Fusion Type A			Sensor Fusion Type B		
	0.05	0.1	0.15	0.05	0.1	0.15
RMSE (m)	0.00111	0.00328	0.00138	0.00555	0.00962	0.019
Accuracy (%)	97.76	96.72	99.08	88.88	90.38	87.32

**Table 4:** Descriptive statistics of tillage depth (m) at different levels of gear selection.

Gear Selection	Max.	Mean	Std.
M3–low	0.188	0.168	0.0088
M3–high	0.194	0.157	0.0013

**Table 5:** Comparison of mean traveling speed (km/h) at different levels of grouped tillage depth.

Gear Selection	Grouped Frequency of Tillage Depth (m)						
	0.14	0.15	0.16	0.17	0.18	0.19	Average
M3–low	5.45 ^A^	5.68 ^B^	5.8 ^C^	5.73 ^BC^	5.51 ^A^	5.44 ^A^	5.69
M3–high	5.74 ^e^	5.9 ^f^	5.63 ^d^	5.36 ^c^	5.03 ^b^	4.1 ^a^	5.58

^A,B,C,a,b,c,d,e,f^ Means within each row with the same combination of letters are not significantly different at *p* < 0.05 according to Duncan’s multiple range tests respectively (capital letters: M3–low gear selection, small letters: M3–high gear selection).

**Table 6:** Comparison of mean draft force (kN) at different levels of grouped tillage depth.

Gear Selection	Grouped Frequency of Tillage Depth (m)						
	0.14	0.15	0.16	0.17	0.18	0.19	Average
M3–low	31.79 ^A^	32.08 ^AB^	32.31 ^B^	32.8 ^C^	33.4 ^D^	34.1 ^E^	32.76
M3–high	32.2 ^a^	32.36 ^ab^	32.54 ^b^	33.49 ^c^	34.43 ^d^	36.51 ^e^	32.91

^A,B,C,D,E,a,b,c,d,e^ Means within each row with the same combination of letters are not significantly different at *p* < 0.05 according to Duncan’s multiple range tests (capital letters: M3–low gear selection, small letters: M3–high gear selection).
